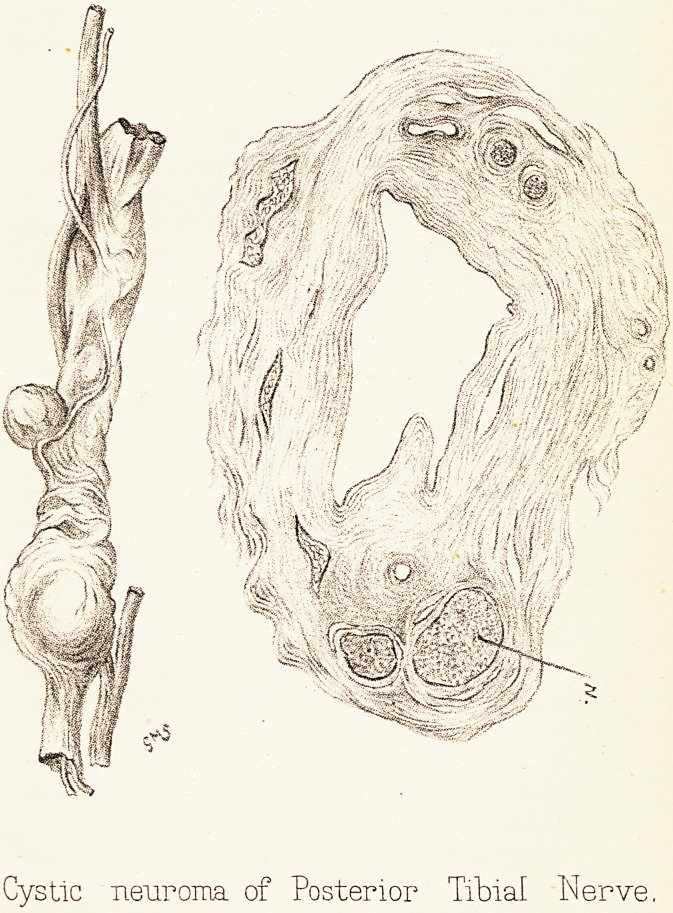# Cystic Neuroma of the Posterior Tibial Nerve

**Published:** 1883-07

**Authors:** J. Fenton Evans

**Affiliations:** House Physician, Bristol Royal Infirmary


### CYSTIC NEUROMA OF THE POSTERIOR TIBIAL NERVE. By J. Fenton Evans, M.B., House Physician, Bristol Royal Infirmary.

Neuromata were first described by Cheselden in 1740, his attention being directed to the cystic variety, although an uncommon form.

According to Paget there are four forms of neuroma (excluding from consideration the somewhat doubtful tumour of nerves, where the true nerve elements are hypertrophied), *i.e.*, cystic, fibrous, fibro-cystic and cancerous, the latter occurring least frequently, and stated by some writers never to originate in nerve trunks, but to invade them from neighbouring structures, "the true neuroma being never malignant."

Neuromata may be idiopathic or traumatic, single or multiple, when traumatic as a rule single. They are more commonly found on cerebro-spinal nerves than on sympathetic.

To the traumatic class belong the fibrous enlargements on the ends of nerves in stumps, often the source of suffering, but as frequently painless, and occurring at some distance from the cicatrix; cases are also recorded of tumours arising in the course of an individual nerve as the result of injury, and producing great pain both above and below the seat of the new fibrous growth, whereas with the single idiopathic tumour the pain is confined to the distribution of the nerve on which it exists.

The idiopathic neuromata may be single or multiple; when single generally very painful, when numerous often undiscovered before death.

I was fortunate enough to meet with a specimen of multiple cystic neuroma on the posterior tibial nerve of a subject in our dissecting room last winter.

On removing the gastrocnemius and soleus muscles with the deep fascia a chain of blueish glistening bodies was to be seen running continuously in the course of the posterior tibial nerve, lobulated and varying in size from a pea to a hazel nut, commencing above in the popliteal space and ending below at the ankle joint, with which the lowest cysts appeared to communicate (Pl. V.).

The nerve increased in size, split up into several bands, some entering the growth at the upper end and some at various points in its course, one trunk running down to the foot independently.

On section the cavities have no connection with each other, and contain a clear albuminous jelly.

Under the microscope the cyst wall is seen to consist

Plate V.

Cystic neuroma of Posterior Tibial Nerve.

of white fibrous tissue, the fibres in many places undergoing mucous degeneration. It seems possible that the formation of the cyst cavity and contents may have been due to a mucous degeneration of the fibres, a fibrous tumour being first formed and gradually breaking down in this manner. The nerve fibres run in two or more bundles through the wall on either side, and appear healthy, the white substance of Schwann not diminished, the axis cylinders staining well.

Mr. Munro Smith has kindly made the drawings for me.

## Figures and Tables

**Figure f1:**